# The efficacy and safety of a duckbill‐type anti‐reflux metal stent as the initial metal stent for distal malignant biliary obstruction in unresectable pancreatic cancer

**DOI:** 10.1002/deo2.205

**Published:** 2023-01-09

**Authors:** Tsuyoshi Takeda, Takashi Sasaki, Yuto Yamada, Takeshi Okamoto, Takafumi Mie, Takaaki Furukawa, Akiyoshi Kasuga, Masato Matsuyama, Masato Ozaka, Naoki Sasahira

**Affiliations:** ^1^ Department of Hepato‐Biliary‐Pancreatic Medicine Cancer Institute Hospital of Japanese Foundation for Cancer Research Tokyo Japan; ^2^ Department of Internal medicine Division of Gastroenterology and Hepatology Toho University Tokyo Japan

**Keywords:** anti‐reflux metal stent, covered metal stent, distal malignant biliary obstruction, pancreatic cancer, recurrent biliary obstruction

## Abstract

**Background:**

The usefulness of duckbill‐type anti‐reflux metal stent (DMS) in self‐expandable metal stent‐naïve pancreatic cancer (PC) patients has not been well‐studied. This study aimed to evaluate the efficacy and safety of DMS in such patients.

**Methods:**

We analyzed consecutive patients with unresectable PC who received a covered metal stent (CMS) as the initial self‐expandable metal stent at our institution. Technical success, functional success, causes of recurrent biliary obstruction (RBO), time to RBO (TRBO), adverse events (AEs), and reintervention rates were compared between DMS and conventional CMS (c‐CMS).

**Results:**

A total of 69 patients were included (DMS: 28, c‐CMS: 41). Technical success, functional success, and AEs were similar between groups. Tumor ingrowth was more common in the DMS group (18% vs. 0%, *p* = 0.009), while non‐occlusion cholangitis tended to be more common in the c‐CMS group (0% vs. 15%, *p* = 0.074). Median time to RBO was similar between groups (276 vs. 273 days, *p* = 0.915). The anti‐reflux valve of DMS was found torn in 56% of patients. Endoscopic reintervention was successful in all cases, despite failed stent removal in 88% of patients in the DMS group.

**Conclusions:**

DMS was not associated with longer time to RBO compared to c‐CMS in self‐expandable metal stent‐naïve patients.

## INTRODUCTION

Distal malignant biliary obstruction (MBO) is a common complication of pancreatic cancer (PC). Endoscopic biliary drainage using self‐expandable metal stents (SEMS) is the standard of care, due to their longer stent patency compared to plastic stents.^1^, ^2^ A recent meta‐analysis reported the superiority of covered metal stents (CMS) over uncovered metal stents for the treatment of distal MBO, especially in patients with PC.^3^ Sludge formation and food impaction resulting from duodenobiliary reflux are major causes of recurrent biliary obstruction (RBO) after CMS placement.^4^, ^5^ Several types of anti‐reflux metal stents (ARMSs) have been developed to prevent duodenobiliary reflux, with conflicting results.^6^, ^7^, ^8^, ^9^, ^10^, ^11^

Duckbill‐type anti‐reflux metal stent (DMS) is a novel fully covered laser‐cut type ARMS. Several studies including ours have reported the efficacy and safety of this stent^12^, ^13^, ^14^, ^15^, ^16^ and DMS was reported to be more effective than conventional CMS (c‐CMS) when used in the preoperative setting^12^ or reinterventions after CMS dysfunction.^15^ However, the superiority of DMS over c‐CMS when used as the initial SEMS for unresectable PC has not been elucidated.

We conducted this retrospective study to evaluate the efficacy and safety of DMS in comparison with c‐CMS as the initial SEMS in patients with unresectable PC.

## METHODS

### Patients

This was a retrospective study of consecutive patients with unresectable PC who received a CMS (DMS or c‐CMS) as the initial SEMS for distal MBO at our institution between January 2020 and November 2021. Only patients who received a CMS across the papilla were included in this study. The following were excluded: (1) patients who had a history of biliary SEMS placement, (2) patients with SEMS placed above the papilla, (3) patients with surgically altered anatomy, and (4) patients with concomitant hilar biliary obstruction. The selection of the type of SEMS was mainly based on the time period in which a given patient underwent initial SEMS placement. In general, c‐CMS was used between January 2020 and November 2020, while DMS was used between December 2020 and November 2021. Written informed consent for the procedure was obtained from every patient. This study was conducted in accordance with the Declaration of Helsinki and was approved by the ethics committee of our institution (Institutional Review Board number: 2021‐GB‐119).

### DMS and c‐CMS

**FIGURE 1:**
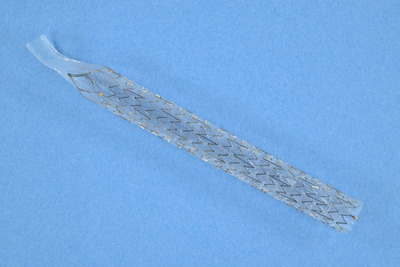
The duckbill‐type anti‐reflux metal stent

The ARMS used in this study was a fully covered laser‐cut type SEMS with a 12.5 mm duckbill‐shaped anti‐reflux valve (ARV) attached to the distal end (Kawasumi Duckbill Biliary Stent; Kawasumi Laboratories Inc., Tokyo, Japan; Figure 1). The ARV is usually closed to prevent duodenobiliary reflux but opens when the bile duct pressure increases. DMSs with diameters of 10 mm and lengths of 60 or 80 mm were used in all patients.

The c‐CMS used in this study was a fully covered braided type SEMS (HANAROSTENT Biliary; M.I.Tech, Seoul, Korea). All stents had diameters of 10 mm and lengths of 60, 70, or 80 mm.

### Endoscopic interventions

All patients underwent endoscopic retrograde cholangiopancreatography (ERCP) using a therapeutic duodenoscope (JF260, TJF260, TJF‐Q290V; Olympus Medical Systems, Tokyo, Japan) under conscious sedation. Endoscopic sphincterotomy was generally performed before SEMS placement and prophylactic rectal nonsteroidal anti‐inflammatory drugs (NSAIDs) were used at the discretion of the endoscopist. Some of the patients had previously undergone plastic stent placement at the referring institution. A SEMS was deployed under fluoroscopic and endoscopic guidance in the first session when the diagnosis of PC had already been confirmed and cholangitis was not present, otherwise, an endoscopic nasobiliary drainage (ENBD) tube was placed in advance and a SEMS was deployed in the next session. Before December 2020, we performed ERCP first and subsequently performed endoscopic ultrasound‐guided fine‐needle aspiration as needed in obstructive jaundice cases without pathological confirmation of cancer. From December 2020 onward (when DMS was generally used), we switched to a strategy of performing endoscopic ultrasound‐guided fine‐needle aspiration before ERCP unless the patient's condition made it preferable to perform ERCP first. Achieving a pathological diagnosis of cancer before ERCP allowed for SEMS placement in a single session, instead of requiring biliary drainage with a plastic stent or ENBD before SEMS placement. The length of SEMS was selected based on cholangiographic findings, and the distal end of the metal part of DMS was placed 5–10 mm below the papilla to completely expose the ARV into the duodenum.

### Outcome measures

Outcomes of SEMS placement were generally defined according to Tokyo Criteria 2014.^17^ In this study, non‐occlusion cholangitis was considered RBO when endoscopic biliary drainage was required to treat cholangitis, while it was considered an adverse event (AE) when it improved conservatively. The primary outcome was time to RBO (TRBO), which was defined as the time from stent placement until RBO occurrence. Patients who were lost to follow‐up or alive at the end of the study period, underwent stent removal due to AEs, underwent conversion surgery, or died without RBO were treated as censored cases at the time of the last follow‐up, stent removal, conversion surgery, or death, respectively. The secondary outcomes were technical success, functional success, causes of RBO, AEs, and overall survival (OS). Technical success was defined as the successful deployment of a SEMS at the intended location, while functional success was defined as a 50% decrease in, or normalization of, serum bilirubin level within 14 days after SEMS placement. When serum bilirubin level was normal at the time of SEMS placement due to prior biliary drainage, functional success was defined as no exacerbation of serum bilirubin level after SEMS placement. The severity of AEs was graded according to the American Society of Gastrointestinal Endoscopy lexicon guidelines.^18^ The duodenal invasion was diagnosed based on the endoscopic findings at the time of SEMS placement. The amount of ascites was evaluated using the most recent computed tomography scan before SEMS placement and was categorized according to the Japanese Classification of Gastric Carcinoma^19^: none, ascites undetected by computed tomography; mild, ascites localized in only one area such as the pelvic cavity; moderate, ascites neither mild nor severe; and severe, ascites throughout the abdominal cavity. Follow‐up data was confirmed until September 30, 2022.

### Statistical analysis

Continuous and categorical variables are expressed as medians with ranges and absolute numbers with proportions, respectively. Categorical variables were compared using the Chi‐square test or Fisher's exact test as appropriate, while continuous variables were compared using the Mann‐Whitney U test. Time to RBO and OS were estimated using the Kaplan‐Meier method and were compared using the log‐rank test. The cumulative incidence of RBO was estimated using the competing risk analysis and was compared using Gray's test.^20^ Stent removal due to AEs or conversion surgery and death without RBO were considered competing events. *p‐*Values < 0.05 were considered statistically significant. All statistical analyses were carried out using the EZR software version 1.40.^21^

## RESULTS

### Patient characteristics

A total of 69 patients were included in this study (28 and 41 patients in the DMS and c‐CMS groups, respectively). The baseline and procedural characteristics of the two groups are summarized in Table 1. A significantly lower proportion of patients underwent biliary drainage before SEMS placement in the DMS group (64% vs. 95%, *p* = 0.002). Other baseline characteristics including duodenal invasion, concomitant duodenal metal stent, presence of moderate to severe ascites, and peritoneal dissemination were not different between the two groups. Stents used in each group were generally 8 cm in the DMS group and 7 cm in the c‐CMS group. Endoscopic sphincterotomy was performed before SEMS placement in all patients, except for one patient in the c‐CMS group who underwent endoscopic papillary balloon dilation because of a previously deployed duodenal metal stent placed across the papilla. Prophylactic NSAIDs were administered more frequently in the DMS group (75% vs. 29%, *p* < 0.001).

**TABLE 1:** Baseline and procedural characteristics of patients who received duckbill‐type anti‐reflux metal stent (DMS) or conventional covered metal stent (c‐CMS) as the initial self‐expandable metal stent (SEMS) for distal malignant biliary obstruction

		**DMS** ***n* = 28**	**c‐CMS** ***n* = 41**	***p*‐Value**
Age (years)		67 (41–76)	67 (46–78)	0.399
Sex	Male	12 (43%)	19 (46%)	0.810
ECOG PS	0/1/2	21 (75%)/6 (21%)/1 (4%)	25 (61%)/ 12 (29%)/4 (10%)	0.442
Tumor status				0.793
Locally‐advanced		8 (29%)	14 (34%)	
Metastatic		20 (71%)	27 (66%)	
Duodenal invasion		6 (21%)	6 (15%)	0.527
Co‐existing duodenal metal stent		0	2 (5%)	0.511
Moderate to severe ascites		0	3 (7%)	0.266
Peritoneal dissemination		3 (11%)	3 (7%)	0.681
Post‐cholecystectomy		1 (4%)	3 (7%)	0.641
Tumor involvement in OCD*		4 (15%)	1 (3%)	0.151
Tumor involvement in PD		24 (86%)	39 (95%)	0.214
Prior history of biliary drainage with ENBD or plastic stent		18 (64%)	39 (95%)	0.002
Chemotherapy after SEMS placement		25 (89%)	35 (85%)	0.729
Stent diameter (mm)	10	28 (100%)	41 (100%)	>0.999
Stent length (cm)	6/7/8	11 (39%)/0/17 (61%)	14 (34%)/23 (56%)/4 (10%)	˂0.001
Papillary intervention before SEMS placement				>0.999
Endoscopic sphincterotomy		28 (100%)	40 (98%)	
Endoscopic papillary balloon dilation		0	1 (2%)	
Prophylactic rectal NSAIDs use		21 (75%)	12 (29%)	˂0.001

Continuous variables are expressed as median (range) and categorical variables are expressed as absolute numbers (proportions).

*Denominators adjusted to exclude four patients who underwent cholecystectomy (one patient and three patients in the DMS and c‐CMS groups, respectively).

Abbreviations: c‐CMS, conventional covered metal stent; DMS, duckbill‐type anti‐reflux metal stent; ECOG, Eastern Cooperative Oncology Group; ENBD, endoscopic nasobiliary drainage; NSAIDs, non‐steroidal anti‐inflammatory drugs; OCD, the orifice of the cystic duct; PD, pancreatic duct; PS, performance status; SEMS, self‐expandable metal stent.

### Outcomes of SEMS

Table 2 summarizes the outcomes of each SEMS. Technical and functional success rates were not different between the two groups.

**TABLE 2:** Outcomes of initial self‐expandable metal stent (SEMS) placement for distal malignant biliary obstruction

	**DMS** ***n* = 28**	**c‐CMS** ***n* = 41**	***p*‐Value**
Technical success	28 (100%)	41 (100%)	>0.999
Functional success	27 (96%)	38 (93%)	0.641
Adverse events*	4 (14%)	3 (7%)	0.430
Pancreatitis	4 (14%)	2 (5%)	0.214
Cholecystitis	1 (4%)	0	0.406
Non‐occlusion cholangitis	0	1 (2%)	>0.999
Causes of recurrent biliary obstruction			
Occlusion	9 (32%)	9 (22%)	0.408
Sludge	4 (14%)	7 (17%)	>0.999
Food impaction	0	1 (2%)	>0.999
Tumor ingrowth	5 (18%)	0	0.009
Tumor overgrowth	0	1 (2%)	>0.999
Migration	3 (11%)	4 (10%)	>0.999
Inward migration	0	0	>0.999
Outward migration	3 (11%)	4 (10%)	>0.999
Complete migration	3 (11%)	3 (7%)	0.681
Incomplete migration	0	1 (2%)	>0.999
Non‐occlusion cholangitis	0	6 (15%)	0.074
Total	12 (43%)	19 (46%)	0.810

Categorical variables are expressed as absolute numbers (proportions).

*One patient in the DMS group developed both pancreatitis and cholecystitis at different times.

Abbreviations: c‐CMS, conventional covered metal stent; DMS, duckbill‐type anti‐reflux metal stent; SEMS, self‐expandable metal stent.

AEs occurred in four patients in the DMS group (moderate pancreatitis: 3, moderate pancreatitis and cholecystitis: 1) and three patients in the c‐CMS group (moderate pancreatitis: 2, moderate non‐occlusion cholangitis: 1; 14% vs. 7%, *p* = 0.430). Of the five patients who developed pancreatitis, four patients underwent SEMS removal on the following day (two patients in each group).

Overall RBO rates were not significantly different between the two groups (43% vs. 46%, *p* = 0.810). Tumor ingrowth was more frequently observed in the DMS group (18% vs. 0%, *p* = 0.009), while non‐occlusion cholangitis tended to be more frequent in the c‐CMS group (0% vs. 15%, *p* = 0.074). Of the five patients who presented with tumor ingrowth in the DMS group, tumor ingrowth was evident at a median follow‐up period of 202 days.

**FIGURE 2:**
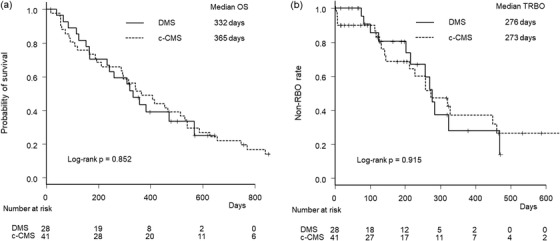
Kaplan‐Meier curves by stent group. (a) Overall survival. (b) Time to recurrent biliary obstruction. DMS, duckbill‐type anti‐reflux metal stent; c‐CMS, conventional covered metal stent; OS, overall survival; RBO, recurrent biliary obstruction; TRBO, time to recurrent biliary obstruction.

**FIGURE 3:**
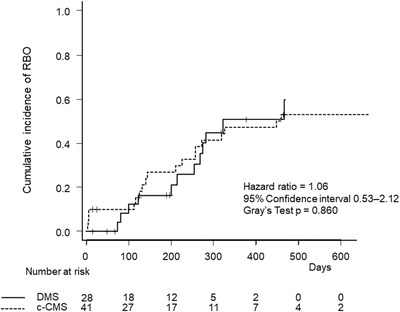
Cumulative incidence of recurrent biliary obstruction by stent group. DMS, duckbill‐type anti‐reflux metal stent; c‐CMS, conventional covered metal stent; RBO, recurrent biliary obstruction.

Kaplan‐Meier curves of OS and TRBO are shown in Figure 2. Both median OS (332 days vs. 365 days, *p* = 0.852) and TRBO (276 days vs. 273 days, *p* = 0.915) were similar between the two groups. The cumulative incidence of RBO was also not different between the two groups (hazard ratio 1.06, 95% confidence interval, 0.53–2.12, *p* = 0.860), even when accounting for competing risks (Figure 3).

### Reintervention after RBO

Twelve patients in the DMS group experienced RBO, of which nine resulted from stent occlusion and three from complete distal stent migration. At the time of reintervention, the ARV was found torn in five of the nine patients (56%) with stent occlusion. Stent removal was attempted in eight patients, but was successful in only one patient (13%). Of the seven unsuccessful cases, tumor ingrowth was present in five and DMS was torn during the removal attempt in two. Biliary cannulation was achieved in all seven unsuccessful cases through the stent mesh (one patient) or the torn ARV of DMS (three patients), or by trimming the distal part of DMS (three patients) using argon plasma coagulation and/or a loop cutter (Olympus Medical Systems). Reintervention was successfully performed in all 12 patients. Seven patients whose stents could not be removed underwent CMS placement in a stent‐in‐stent method. Three patients underwent CMS replacement after successful stent removal or stent migration, one underwent plastic stent placement in a stent‐in‐stent method (stent removal of the initial SEMS was not attempted in this patient due to a giant duodenal ulcer), and one underwent plastic stent placement after successful stent removal.

Nineteen patients in the c‐CMS group experienced RBO, of which nine resulted from stent occlusion, four from stent migration, and six from non‐occlusion cholangitis. Stent removal was successful in all attempted cases (stent removal was not attempted in one patient due to a combined type 2 biliary and duodenal obstruction). Reintervention was successfully performed in all 19 patients. Fourteen patients underwent CMS replacement, one underwent simultaneous duodenal stenting and endoscopic ultrasound‐guided choledochoduodenostomy, one underwent endoscopic ultrasound‐guided hepaticogastrostomy, one underwent plastic stent placement, and two underwent endoscopic nasobiliary drainage.

## DISCUSSION

This retrospective study evaluated the efficacy and safety of DMS in comparison with c‐CMS for distal MBO in SEMS‐naïve unresectable PC patients. DMS was not associated with longer TRBO compared to c‐CMS (276 vs. 273 days, *p* = 0.915). Reasons for RBO differed between the two groups, with a higher rate of tumor ingrowth in the DMS group (18% vs. 0%, *p* = 0.009) and a tendency of a higher rate of non‐occlusion cholangitis in the c‐CMS group (0% vs. 15%, *p* = 0.074). Overall rates of AEs were similar between the two groups, with acute pancreatitis being the most common AE. The ARV of DMS was found torn in five of the nine patients (56%) who experienced RBO due to stent occlusion. Although stent removal of DMS was unsuccessful in most patients (88%) mainly as a result of tumor ingrowth, endoscopic reintervention was successful in every patient using the stent‐in‐stent method.

Covered SEMS, which was developed to prevent stent occlusion due to tumor ingrowth, has been considered the standard first‐line treatment for distal MBO in unresectable PC ^3^. Sludge formation and stent migration remain the main causes of stent dysfunction of CMSs.^3^ Development of stents with anti‐migration properties (e.g., flared ends)^22^, ^23^ has contributed to the reduction of stent migration. Several braided‐type ARMSs with various types of ARVs ^6^, ^7^, ^8^, ^9^, ^10^, ^11^ were developed to prevent duodenobiliary reflux, in turn reducing the risks of sludge formation and food impaction. In a recent meta‐analysis, braided‐type ARMSs were reported to have a lower rate of stent occlusion but a higher rate of stent migration compared to conventional SEMSs,^24^ highlighting the need for the development of ARMSs with effective anti‐migration systems. The DMS used in this study is the first laser‐cut type ARMS, which may have longer TRBO compared to braided‐type ARMS due to the presumed lower risk of stent migration.^25^ Indeed, the rate of DMS migration in this study (11%) was much lower than that of braided‐type ARMS with a long funnel‐type valve (31%) which was reported by Hamada et al.^11^ Evidence to date suggests that DMS offers longer TRBO compared to c‐CMS, especially when used in the preoperative setting^12^ or reinterventions after CMS dysfunction.^15^ However, evidence is limited regarding the efficacy of DMS in comparison with c‐CMS in SEMS‐naïve unresectable patients.

In the present study, DMS was not associated with longer TRBO compared to c‐CMS in SEMS‐naïve unresectable PC patients. Although the rate of stent migration was similar between the two groups (11% vs. 10%) and the rate of non‐occlusion cholangitis was lower in the DMS group (0% vs. 15%, *p* = 0.074), the rate of stent occlusion was somewhat higher in the DMS group (32% vs. 22%, *p* = 0.408), resulting in similar overall RBO rates between the two groups. The negative results of our study could be due to several reasons including patient characteristics and the durability of the ARV and covered membrane. First, patients who would most benefit from ARMS are those who are at high risk of duodenobiliary reflux, including those with duodenal invasion and those who have indwelling duodenal stents.^26^, ^27^ The proportion of such patients was low in this study, indicating that only a few patients benefited from the use of ARMS. Second, the ARV of DMS was found to be torn in 56% of RBO cases, suggesting that the present ARV of DMS may not be durable enough to prevent duodenaobiliary reflux for a long period of time. Since a previous in vitro study showed that a duodenal pH environment caused a morphological change of ARV,^27^ the development of a new ARV that is not affected by the duodenal pH environment might be necessary to improve the durability of the ARV. Third, the rate of tumor ingrowth was high in this study (18%) compared to previous studies using DMS.^12^, ^13^, ^15^, ^28^ This could be explained by the difference in the proportion of SEMS‐naïve patients and the duration of the indwelling period of SEMS.^29^ We speculated that the external force applied to the covered membrane of a laser‐cut type SEMS may become strong enough to cause tumor ingrowth, especially when used in SEMS‐naïve patients with a long indwelling time. Thus, modification of the design of the covered membrane might also be required to further prolong the TRBO of DMS, especially when used in SEMS‐naïve PC patients.

Reintervention after RBO of the initial CMS is becoming an important issue, as the prognosis of PC patients has improved due to recent advances in chemotherapy. Although stent removal of DMS was unsuccessful in a majority of attempted cases in this study, biliary cannulation was successfully achieved through the stent mesh or the torn ARV of DMS, or by trimming the distal part of DMS using argon plasma coagulation and/or a loop cutter. As a result, endoscopic reintervention was successful in every patient. Conversion to endoscopic ultrasound‐guided intervention is also a possible salvage method after RBO of DMS.

The current study has several limitations. This was a retrospective study from a single institution with a limited sample size. Although consecutive patients with unresectable PC were included in this study, selection bias cannot be completely avoided. Patient characteristics were different in some aspects including the presence of prior biliary drainage and prophylactic rectal NSAID use. This was mainly due to changes in the treatment strategy for undiagnosed PC patients with obstructive jaundice; endoscopic ultrasound‐guided fine‐needle aspiration was more frequently performed before biliary drainage in the DMS group. The exact rate and timing of tearing of the ARV are unknown because we only evaluated patients who experienced RBO.

In conclusion, DMS was not associated with an extended duration of TRBO compared to c‐CMS in SEMS‐naïve patients. Further modifications of the present DMS, including the design of the ARV and the covered membrane, may be needed before using this stent as the first‐line biliary drainage for distal MBO in unresectable PC patients.

## CONFLICT OF INTEREST

None.
